# When ‘Mucocele’ Isn’t Just a Mucocele: Diagnostic Pathways and Surgical Management of Appendiceal Mucinous Lesions in a 10-Year Cohort

**DOI:** 10.3390/medicina62050847

**Published:** 2026-04-29

**Authors:** Diana Maria Orzata, Adrian-Iosif Moldoveanu, Gabriel Veniamin Cozma, Radu Gheorghe Dan, Ovidiu Alexandru Mederle, Flavia Zara, Raluca Maria Closca, Laurentiu Vasile Sima

**Affiliations:** 1 Doctoral School, “Victor Babeș” University of Medicine and Pharmacy, 300041 Timisoara, Romania; diana.orzata@umft.ro (D.M.O.); adrian.moldoveanu@umft.ro (A.-I.M.); 2 Department of Surgery I, “Victor Babeș” University of Medicine and Pharmacy, 300041 Timisoara, Romania; gabriel.cozma@umft.ro (G.V.C.); sima.laurentiu@umft.ro (L.V.S.); 3 Center for Hepato-Biliary-Pancreatic Surgery, “Victor Babeș” University of Medicine and Pharmacy, 300041 Timisoara, Romania; 4 Emergency Discipline, Department of Surgery, “Victor Babeș” University of Medicine and Pharmacy, 300041 Timisoara, Romania; mederle.ovidiu@umft.ro; 5 Department of Surgery, Multidisciplinary Centre for Research, Evaluation, Diagnosis and Therapies in Oral Medicine, “Victor Babeș” University of Medicine and Pharmacy, 300041 Timisoara, Romania; 6 Department of Microscopic Morphology, “Victor Babeș” University of Medicine and Pharmacy, 300041 Timisoara, Romania; flavia.zara@umft.ro (F.Z.); raluca.moaca@umft.ro (R.M.C.); 7 Department of Pathology, Emergency City Hospital, 300254 Timisoara, Romania

**Keywords:** appendiceal mucocele, appendiceal mucinous lesions, LAMN, HAMN, histopathology, surgical management, diagnostic pathway, clinical utility

## Abstract

*Background and Objectives*: The term “appendiceal mucocele” is widely used in clinical practice, yet it remains diagnostically incomplete because it represents a preoperative clinical and imaging label rather than a definitive diagnosis. We examined the diagnostic and therapeutic pathway of patients managed under this working label over a 10-year period and assessed how final histopathology influenced subsequent management. *Materials and Methods*: We performed a retrospective single-center cohort study including patients with appendiceal mucinous lesions managed between January 2016 and December 2025. Eligible cases were identified from the institutional histopathology registry and classified on final histopathology as non-neoplastic mucoceles or neoplastic mucinous lesions, including low-grade and high-grade appendiceal mucinous neoplasms. Data were extracted on CT use, surgical strategy, margin status, extra-appendiceal/serosal mucin, chemotherapy, recurrence, and follow-up. *Results*: Eighteen patients met the eligibility criteria. Final histopathology showed two distinct endpoints: non-neoplastic mucoceles and neoplastic mucinous lesions. CT was the dominant preoperative investigation and most often supported the working impression of mucocele or suspected mucinous appendiceal lesion, although some neoplastic cases entered the pathway as presumed appendicitis or perforation. Appendectomy was the dominant index procedure, whereas primary right hemicolectomy was uncommon. Completion right hemicolectomy was performed only in the neoplastic subgroup, and management decisions were driven by a limited number of actionable histopathologic features, particularly margin status and extra-appendiceal/serosal mucin. *Conclusions*: This study highlights a real-world diagnostic and therapeutic pathway in which the term “mucocele” represents a preoperative label rather than a definitive diagnosis. Histopathology appeared to represent the key step in this pathway, rather than the preoperative imaging impression. Given the small sample size, these findings should be interpreted as descriptive and hypothesis-generating. In practical terms, suspected appendiceal mucocele should be approached through a standardized pathway involving safe index resection, structured pathology reporting using modern terminology, and risk-adapted escalation and follow-up when higher-risk features are present.

## 1. Introduction

Appendiceal “mucocele” is a descriptive clinical and imaging label for an abnormal mucus-related dilatation of the appendix rather than a single histologic diagnosis. In imaging-based practice, the term is used when a cystic, blind-ending tubular structure contiguous with the cecum is identified, but the underlying cause may be non-neoplastic or neoplastic. Definitive classification, and therefore risk stratification, relies on histopathologic evaluation [1].

Appendiceal tumors are rare and heterogeneous entities, identified in approximately 1–2% of appendectomy specimens, and may range from benign lesions to mucinous neoplasms and adenocarcinomas. Their clinical presentation is variable, from incidental findings to acute appendicitis-like syndromes, which contributes to diagnostic uncertainty at initial evaluation [2,3,4].

Over the past decade, clearer terminology and standardized reporting have refined this spectrum, particularly through the consistent use of low-grade appendiceal mucinous neoplasm (LAMN) and high-grade appendiceal mucinous neoplasm (HAMN). Contemporary classification frameworks and diagnostic reviews emphasize that these entities differ from simple mucoceles and require structured pathologic assessment, including documentation of features relevant to staging and downstream management [5,6,7,8].

From a management standpoint, current recommendations support a diagnostic–therapeutic pathway centered on safe resection with avoidance of rupture/spillage, followed by histopathology-driven decisions. Margin status and the presence of extra-appendiceal/serosal mucin are commonly management-relevant findings that may inform multidisciplinary discussion, surveillance, or escalation in selected cases [9,10,11,12].

Clinically, appendiceal mucinous lesions are uncommon and may be discovered incidentally or present through acute surgical pathways mimicking appendicitis or obstruction. Large appendectomy series report low detection rates of appendiceal neoplasms, and systematic analyses suggest that occult malignancy may be encountered in inflammatory appendiceal presentations [2,3,4].

In this context, we aimed to describe a real-world diagnostic and therapeutic pathway in patients managed under the preoperative label of “mucocele,” focusing on how final histopathology influences subsequent management decisions. Given the rarity of these lesions, our study is intended as a descriptive, pathway-oriented analysis rather than a hypothesis-testing investigation.

## 2. Materials and Methods

### 2.1. Study Design and Surgical Volume

This is a retrospective, single-center cohort study including patients operated between January 2016 and December 2025. Over the same interval, our unit performed 2492 appendectomies and 704 right hemicolectomies, figures reported to contextualize the frequency of appendiceal mucinous lesions within routine surgical activity.

### 2.2. Case Retrieval and Eligibility

Eligible cases were retrieved from the institutional histopathology registry by screening appendiceal specimens described as mucocele and/or mucinous appendiceal lesions, including reports using the diagnostic terms LAMN and HAMN. Each candidate record was subsequently verified by review of the pathology report and the corresponding clinical file.

We included only appendiceal mucinous lesions confirmed on final histopathology and managed during the study period, classified into the following:

• non-neoplastic mucocele;
• neoplastic mucinous lesions (LAMN/HAMN).

We excluded cases with other appendiceal tumors (e.g., neuroendocrine tumors, appendiceal adenocarcinoma) and cases without definitive histopathologic confirmation.

A total of 3196 histopathology reports were reviewed, corresponding to all appendectomies and right hemicolectomies performed during the study period. Of these, 3178 were non-mucinous, including 3128 cases of acute appendicitis, 31 cases of appendicitis chronica obliterans, 6 neuroendocrine tumors, 6 appendiceal adenocarcinomas, and 7 cases with incomplete data. The remaining 18 cases met the inclusion criteria and constituted the final study cohort of appendiceal mucinous lesions.

### 2.3. Variables and Operational Definitions

Data were extracted from operative notes, discharge summaries, radiology reports, pathology reports, and oncology records when available.

Index procedure and completion surgery. The index procedure was defined as the first operation performed for the presenting episode. Index procedures were recorded as appendectomy (open or laparoscopic) or primary right hemicolectomy. Completion right hemicolectomy was defined as a right hemicolectomy performed after an initial appendectomy as a consequence of histopathologic findings and subsequent surgical decision-making. When completion surgery was performed, the interval from appendectomy to completion was recorded in weeks.

Diagnostic pathway (CT). CT use was recorded as performed/not performed. When performed, the radiology report impression was categorized as follows:

• mucocele/suspected mucinous lesion;
• appendicitis/perforation without mucocele suspicion;
• obstruction;
• other/indeterminate.

Imaging variables were used to describe the diagnostic pathway and were not analyzed as diagnostic accuracy measures for histologic subtype. This study was not designed as a diagnostic accuracy study, and CT findings were interpreted in a descriptive, pathway-oriented context.

Histopathology. Final histology was recorded as non-neoplastic mucocele, LAMN, HAMN, or combined LAMN/HAMN (when reported). The combined category was used for specimens showing distinct areas with conventional LAMN morphology together with separate foci displaying HAMN-type high-grade cytologic atypia within the same appendiceal lesion. Margin status after the index procedure was recorded as R0 or R1. For patients undergoing completion surgery, final margin status after completion was recorded separately. Extra-appendiceal mucin (including periappendiceal mucin extravasation and/or serosal mucin deposits) was recorded as present when documented in the pathology report and as absent otherwise, in accordance with institutional reporting practice. The absolute lymph node yield was not analyzed because it was not consistently reported.

Systemic therapy, recurrence, follow-up. Chemotherapy was recorded as yes/no when documented. Recurrence was recorded only when explicitly documented clinically and/or radiologically; when present, the site was specified. Follow-up was defined as the time from the index procedure to the last documented clinical contact within available records. Structured follow-up was not uniformly available for non-neoplastic cases, and recurrence data in this subgroup should therefore be interpreted with caution.

### 2.4. Outcomes

The primary outcome was the proportion of neoplastic mucinous lesions (LAMN/HAMN) among patients managed under the working clinical/imaging label of “mucocele”/appendiceal mucinous lesion in routine practice.

Secondary outcomes were: CT utilization and report impression categories; index versus completion surgical strategy (including time to completion hemicolectomy); R1 margins after appendectomy; presence of extra-appendiceal/serosal mucin; chemotherapy use; and documented recurrence.

### 2.5. Statistical Analysis

Continuous variables are presented as median (interquartile range, IQR) and categorical variables as number (percentage). Given the small sample size, all analyses were considered descriptive and exploratory, and no formal inferential conclusions were intended. Normality of continuous variables was assessed using the Shapiro–Wilk test. As most variables did not follow a normal distribution, they are reported using median and interquartile range (IQR), and non-parametric tests were applied. Comparisons between non-neoplastic and neoplastic (LAMN/HAMN) groups were performed using Fisher’s exact test for categorical variables and the Mann–Whitney U test for continuous variables. Statistical analysis was performed using IBM SPSS Statistics (version 26.0; IBM Corp., Armonk, NY, USA).

## 3. Results

### 3.1. Study Cohort and Case Yield

Between January 2016 and December 2025, 18 patients met the eligibility criteria for appendiceal mucinous lesions (10-year cohort). Over the same period, 2492 appendectomies and 704 right hemicolectomies were performed in our unit. Of the 18 included cases, 16 were managed with appendectomy as the index procedure, corresponding to 0.64% (16/2492) of all appendectomies performed during the study interval. Two patients underwent primary right hemicolectomy as the index operation.

**Figure 1:**
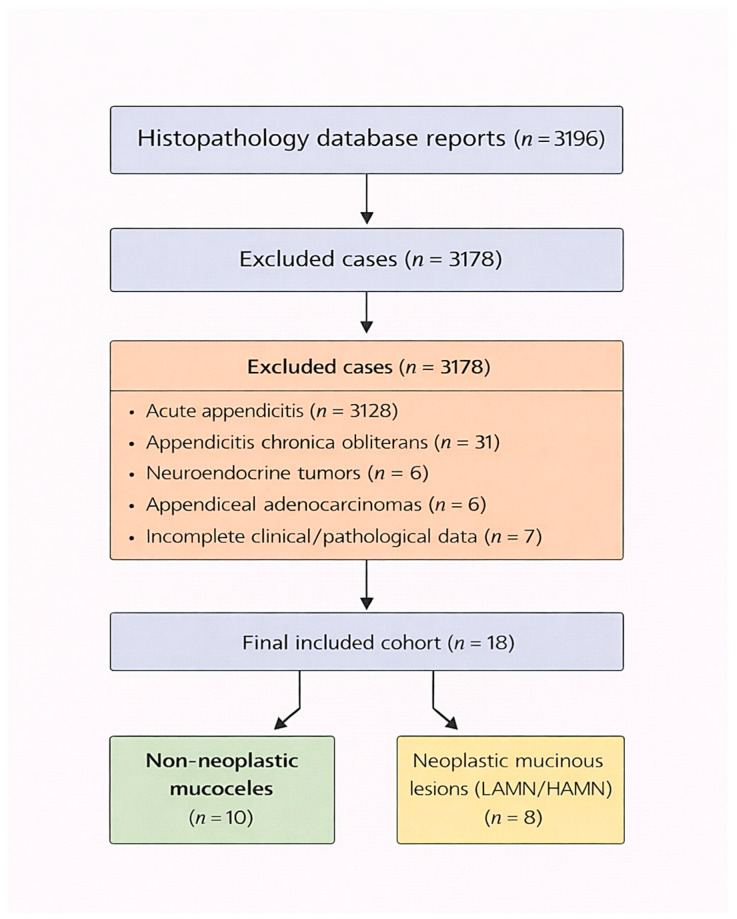
Flowchart of case selection, exclusions, and final cohort classification.

On final histopathology, 10/18 (55.6%) were classified as non-neoplastic mucoceles, and 8/18 (44.4%) as neoplastic mucinous lesions (LAMN/HAMN) (Figure 1).

### 3.2. Baseline Characteristics and Presentation

The overall median age was 64.5 years (IQR 62.5–66.5), and 12/18 (66.7%) patients were female. Differences in presentation were observed between groups: all neoplastic cases presented through an emergency pathway (8/8, 100.0%), whereas most non-neoplastic mucoceles were identified through non-emergency pathways (8/10, 80.0%). Baseline characteristics and presentation patterns are summarized in Table 1.

### 3.3. Diagnostic Pathway (CT Use and Report Impression)

CT was performed in 16/18 (88.9%) patients. Among those with CT, the report impression was: mucocele/suspected mucinous lesion in 12/16 (75.0%), appendicitis/perforation without mucocele suspicion in 2/16 (12.5%), and obstruction in 2/16 (12.5%).

CT utilization differed by subgroup: all non-neoplastic cases had CT (10/10), while CT was not performed in 2/8 neoplastic cases (both presenting with acute appendicitis). Two neoplastic cases had CT interpreted as appendicitis/perforation without suspicion of mucocele.

CT utilization and report impressions are detailed in Table 2.

### 3.4. Surgical Management (Index and Completion Procedures)

The index operation was appendectomy in 16/18 (88.9%) and primary right hemicolectomy in 2/18 (11.1%) (both in the neoplastic group). No intraoperative rupture/spillage of the appendix was documented in this cohort.

Among neoplastic lesions, completion right hemicolectomy after appendectomy was performed in 4/8 (50.0%) patients (and in 4/6 (66.7%) of neoplastic cases initially managed by appendectomy). The interval from appendectomy to completion surgery ranged from 4 to 6 weeks (median 5.5 weeks). The remaining neoplastic cases were treated either with appendectomy alone (when R0 and no escalation was pursued) or with primary right hemicolectomy.

Index and completion surgical strategies are summarized in Table 3.

### 3.5. Histopathology and Actionable Findings

Final histopathology showed LAMN in 4 cases, HAMN in 1 case, and combined LAMN/HAMN in 3 cases. In these three combined cases, both low-grade and focal high-grade components were identified within the same specimen. In the non-neoplastic group, underlying pathological substrates included simple mucoceles and mucoceles associated with chronic inflammatory changes, including granulomatous inflammation.

Margin status after the index procedure was R1 in 4/18 (22.2%), and all R1 margins occurred in the neoplastic group (4/8, 50.0%). Completion right hemicolectomy was subsequently performed in these cases, achieving R0 final margins.

Extra-appendiceal/serosal mucin was documented in 4/18 (22.2%) cases, all within the neoplastic subgroup (4/8, 50.0%). In selected neoplastic cases, extra-appendiceal mucin was associated with epithelial components, and advanced local disease (pT4a) was documented.

Actionable histopathologic findings are presented in Table 4.

**Figure 2:**
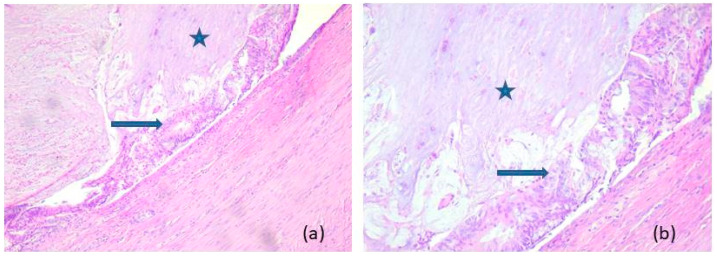
Non-neoplastic appendiceal mucocele: microscopic aspects in hematoxylin–eosin staining, (**a**) 10×, (**b**) 20×. The appendix shows atrophied mucosa (arrow) compressed by basophilic mucin (star).

**Figure 3:**
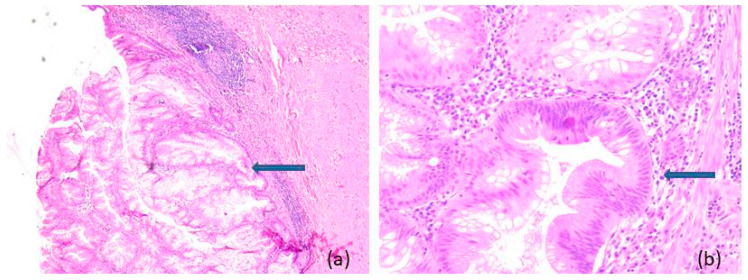
Low-Grade Appendiceal Mucinous Neoplasms (LAMN): microscopic aspects in hematoxylin-eosin staining, (**a**) 5×, (**b**) 20×: villous proliferation of mucinous epithelial cells, with abundant apical mucin, elongated nuclei, and low-grade nuclear atypia (arrow).

**Figure 4:**
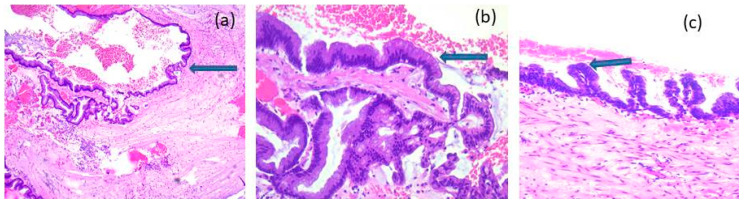
High-Grade Appendiceal Mucinous Neoplasms (HAMN): microscopic aspects in hematoxylin-eosin staining, (**a**) 10×, (**b**) 20×, (**c**) 20×: proliferation of epithelial cells, with depletion of apical mucin, elongated or oval nuclei, and high-grade cytological features (arrow).

Representative microscopic aspects of non-neoplastic appendiceal mucocele are shown in Figure 2, whereas representative features of LAMN and HAMN are shown in Figure 3 and Figure 4, respectively.

### 3.6. Systemic Therapy, Recurrence, and Follow-Up

Systemic chemotherapy was administered in 2/18 (11.1%) patients, both in the neoplastic subgroup.

A single recurrence was documented (1/18, 5.6%; 1/8, 12.5% within neoplastic lesions), presenting as abdominal wall mucinous carcinomatosis approximately 12 months after completion hemicolectomy; it was resected with negative margins, and the patient continued systemic therapy.

Structured oncologic follow-up was available for all neoplastic cases, with documented follow-up ranging from 12 to 84 months (median 27 months). In contrast, non-neoplastic mucoceles did not undergo standardized surveillance, and outcome data in this subgroup are therefore limited.

## 4. Discussion

### 4.1. Principal Findings

In this 10-year cohort, “appendiceal mucocele” was used as a preoperative clinical/imaging label rather than a definitive diagnosis, with final classification based on histopathology in line with current frameworks [1,5,6,7,8].

Both non-neoplastic mucoceles and neoplastic mucinous lesions were identified, reflecting the pathway-based inclusion of cases rather than a neoplasm-focused registry. Appendectomy-based series likewise show that appendiceal neoplasms are uncommon overall and that histologic composition varies according to inclusion criteria and clinical setting, while inflammatory appendiceal presentations may still conceal underlying malignancy [2,3,4].

Management-relevant pathology findings were observed predominantly in the neoplastic subgroup, including R1 margins after appendectomy, extra-appendiceal/serosal mucin, and completion right hemicolectomy. This is consistent with guideline and consensus documents that frame downstream decisions around histology, margin status, and mucin beyond the appendix rather than around the preoperative label alone [9,10,11,12].

Given the small sample size, these findings should be interpreted as descriptive and hypothesis-generating, providing a pathway-oriented overview of how the mucocele label resolves into distinct histologic endpoints and may influence management [13,14,15,16,17].

### 4.2. “Mucocele” as a Label: What Our Cohort Adds

The term “mucocele” should be understood as a preoperative label rather than a definitive diagnosis, with final classification based on histopathology and standardized terminology (LAMN/HAMN) [1,5,6,7,8,15].

Unlike neoplasm-focused series, this cohort reflects a pathway-based inclusion in which patients enter under a mucocele label and are subsequently classified by histopathology, capturing both benign and neoplastic endpoints. This explains the predominance of non-neoplastic mucoceles and is consistent with reports highlighting heterogeneity in final pathology and presentation [10,11,13,16,17].

In this cohort, neoplastic lesions frequently presented through emergency pathways, including cases initially interpreted as acute appendicitis, consistent with evidence that appendiceal neoplasia may be identified only after surgery for presumed inflammatory disease [2,3,4,18,19,20].

The key clinical implication is that the same preoperative label may correspond to different histologic endpoints, with management decisions ultimately guided by histopathology rather than imaging alone [1,5,6,7,8].

Standardized classification and reporting improve communication and management decisions, particularly when extra-appendiceal mucin is present, supporting a pathway that culminates in a definitive histopathologic diagnosis [6,7,8,21].

The identification of mixed LAMN/HAMN morphology in several specimens highlights that appendiceal mucinous neoplasia may show intralesional histologic heterogeneity and underscores the importance of careful sampling and detailed pathology reporting, particularly when focal high-grade areas are present [5,8].

### 4.3. Diagnostic Pathways: What Imaging Can and Cannot Do

In our cohort, CT was the dominant preoperative investigation and most often generated the working label of “mucocele” or “suspected mucinous appendiceal lesion”. This is consistent with radiology-focused overviews that describe CT as the key modality for recognizing a dilated mucus-filled appendix and for documenting complications such as inflammation, perforation, or obstruction [1]. However, CT remains a pathway tool that supports suspicion and operative planning but does not provide definitive etiologic classification or replace histopathology as the diagnostic reference standard [1,5,6,7,8].

Neoplastic lesions did not consistently enter the pathway with a “mucocele” label, with some cases initially interpreted as acute appendicitis or perforation, consistent with evidence that appendiceal neoplasms are often identified only after surgery for presumed inflammatory disease [2,3]. Systematic synthesis of acute appendicitis and inflammatory appendiceal mass further supports a non-zero probability of underlying malignancy, strengthening the argument for structured specimen evaluation and cautious interpretation of purely inflammatory preoperative labels [4].

Appendectomy-based cohorts further show that appendiceal neoplasms are uncommon but may be incidentally identified after surgery, supporting the limitation of imaging-based suspicion in detecting all neoplastic cases [2,3].

This study was not designed to assess CT diagnostic accuracy but to describe how imaging findings distribute within a clinical pathway and relate to final histopathologic endpoints. In our cohort, CT reports of “mucocele” occurred in both non-neoplastic and neoplastic lesions, reinforcing that the same imaging label can map to different etiologies and that the clinically relevant closure of the pathway is the histopathology report with standardized terminology (LAMN/HAMN) and explicit reporting of risk features [5,6,7,8].

Clinical context remains essential, as mucinous neoplasms may present either as acute appendicitis or as cystic appendiceal lesions, reinforcing the heterogeneity of presentation [18,19,20]. Taken together, our cohort supports a practical conclusion consistent with both imaging and pathology frameworks: CT is useful for recognition and operative planning, but definitive etiologic classification and risk stratification require histopathology and standardized reporting [1,5,6,7,8].

### 4.4. Surgical Management and Escalation Pathways

**Figure 5:**
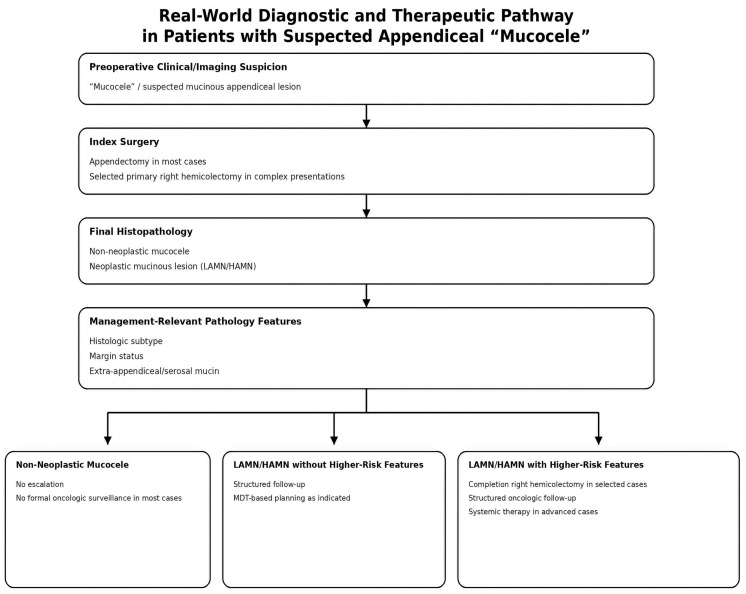
Real-world diagnostic and therapeutic pathway in patients with suspected appendiceal “mucocele”. The figure summarizes the practical sequence observed in our cohort, from preoperative clinical/imaging suspicion to index surgery, final histopathologic classification, and postoperative management. In this framework, “mucocele” functions as a working preoperative label rather than a definitive diagnosis. Subsequent management is primarily determined by histopathology, particularly histologic subtype, margin status, and the presence of extra-appendiceal/serosal mucin.

In this cohort, surgical management followed a pragmatic pathway: an index operation performed under diagnostic uncertainty, followed by histopathology-guided escalation when indicated (Figure 5). This framing is consistent with major guidelines and consensus statements, which emphasize safe resection and downstream decisions anchored in histologic subtype and management-relevant pathology features rather than in the preoperative “mucocele” label [9,10,11].

Appendectomy was the dominant index procedure, while primary right hemicolectomy was uncommon and limited to selected neoplastic cases, reflecting a context-driven approach influenced by intraoperative findings and clinical judgment [11,13]. This is consistent with appendectomy-based evidence showing that neoplasia may be identified after surgery for presumed appendicitis or inflammatory appendiceal mass [2,3,4].

Completion right hemicolectomy after appendectomy was observed in neoplastic mucinous lesions, typically within 4–6 weeks, reflecting a pathway in which definitive classification and risk stratification become available only after histopathology. In this cohort, escalation was associated with a limited number of pathology variables, particularly R1 margin status and the presence of extra-appendiceal/serosal mucin, which represent key decision points in clinical practice and are emphasized in guideline-based management pathways [5,6,7,8,9,10,11].

R1 margins after appendectomy occurred exclusively in the neoplastic subgroup and were followed by completion right hemicolectomy, achieving R0 margins; however, management of R1 margins in LAMN remains debated and may require individualized decision-making [12].

Extra-appendiceal/serosal mucin represents an additional escalation factor, as its presence is associated with more intensive management pathways and is emphasized in modern pathology reporting and staging frameworks [5,6,7,8,9,10,11].

A counterbalance to escalation is that not all mucinous neoplasms follow an aggressive course when confined to the appendix and when high-risk features are absent. Outcome-oriented pathology work has reported excellent outcomes in mucinous neoplasms confined to the appendix, supporting a risk-adapted approach and avoidance of overtreatment when management-relevant high-risk features are not present [22]. This aligns with the broader principle embedded in guideline-based pathways: escalation is driven by concrete pathology variables rather than applied uniformly to all patients carrying the mucocele label [9,10,11].

Operative conduct remains important due to the risk of rupture or spillage; no such events were documented in this cohort, consistent with risk-mitigation principles emphasized in guidance documents [9,10,11,14].

Overall, these findings support a pathology-driven and individualized approach to escalation in appendiceal mucinous lesions, consistent with the heterogeneity described in clinical and guideline-based literature [16,17,23].

### 4.5. Outcomes and Follow-Up

Follow-up in this cohort was strongly conditioned by histologic endpoint, which is typical for pathway-based, real-world series. Structured oncologic follow-up was available for all neoplastic mucinous lesions (LAMN/HAMN), whereas most non-neoplastic mucoceles did not enter formal surveillance pathways. This imbalance limits direct outcome comparisons between groups, and recurrence data in the non-neoplastic subgroup should be interpreted with caution due to the absence of structured follow-up. Risk-adapted follow-up and multidisciplinary team-based decision-making are repeatedly emphasized across major guidance and consensus documents for appendiceal tumors and peritoneal surface malignancy, particularly when mucinous neoplasia is diagnosed [9,10,11]. Narrative overviews of diagnosis, management, and follow-up likewise support tailoring surveillance intensity to histology and risk features rather than to the preoperative label alone [24].

Within the neoplastic subgroup, documented follow-up ranged from 12 to 84 months, and a single recurrence was recorded after completion right hemicolectomy and systemic therapy. Given the small number of neoplastic cases and heterogeneous follow-up durations, recurrence rates should not be interpreted as prognostic estimates. The more appropriate interpretation is pathway-based: recurrence occurred in a context of management-escalation features and illustrates the need for clear documentation of operative history and pathology risk features when planning follow-up. Modern classification and staging discussions emphasize that risk interpretation in appendiceal mucinous disease depends on standardized histology terminology and on explicit reporting of disease beyond the appendix, which directly informs downstream monitoring strategies [5,6,7,8]. Reviews addressing mucinous appendiceal neoplasms and pseudomyxoma peritonei similarly highlight that peritoneal-surface risk is shaped by pathology context and should be managed within structured multidisciplinary frameworks [10,11,17].

A practical implication of our dataset is that the same histopathologic variables that drove escalation also concentrated follow-up intensity in the neoplastic group, particularly margin status and extra-appendiceal/serosal mucin. This supports a conservative statement: in a mucocele-pathway cohort, the follow-up plan is primarily determined after histopathology, not at the time of CT labeling [5,6,7,8,9,10,11]. It also reinforces why reporting standards matter: without explicit documentation of these variables, clinicians may default to either excessive surveillance or inadequate follow-up [5,6,7,8,24].

Systemic therapy was administered in two neoplastic cases. These numbers are too small to support any inference about benefit, and our study was not designed to evaluate chemotherapy efficacy. The role of systemic therapy in appendiceal malignancies has largely been investigated in registry-based analyses that include heterogeneous histologies, often focused on adenocarcinoma, and results are typically influenced by stage, grade, and histologic subtype [25,26]. These datasets provide context for why systemic therapy may be considered in selected appendiceal cancers but should not be extrapolated as evidence of benefit in LAMN/HAMN pathway cohorts such as ours. Contemporary management overviews similarly emphasize histology-specific decision-making and multidisciplinary discussion in mucinous appendiceal disease, especially when extra-appendiceal mucin or high-grade features are present [10,11,13].

Finally, a small but relevant methodological point is staging language. One neoplastic case in our cohort had advanced local staging documented (pT4a). While staging manuals primarily address appendiceal adenocarcinoma, they provide a common language for communicating depth of invasion and local extension, which can be useful when describing complex cases even within mucinous pathways [27]. In the present cohort, staging is used descriptively, while the primary interpretive focus remains on standardized histology terminology and management-relevant pathology features [5,6,7,8].

### 4.6. Comparison with Published Data

Interpreting our results requires aligning like with like, because the published literature on appendiceal mucinous disease is built on different sampling frames. Some studies are appendectomy-incidence cohorts that quantify neoplasms among all appendectomy specimens, whereas others are pathology-led or peritoneal-surface cohorts enriched for mucinous neoplasms and advanced disease [2,3,10,11]. Our study fits a third category: a mucocele-pathway cohort, in which the entry point is a clinical or imaging working label and the endpoint is histopathology-driven classification and management.

Appendectomy-based cohorts consistently show that appendiceal neoplasms are uncommon overall and are often identified only after surgery performed for presumed appendicitis [2,3,4]. This is consistent with the predominance of emergency presentations in neoplastic cases in our cohort. At the same time, neoplasm-enriched series and guideline frameworks emphasize that LAMN and HAMN are not merely “mucoceles,” but distinct entities with specific staging and reporting implications, particularly when mucin extends beyond the appendix [5,6,7,8,10,11].

The predominance of non-neoplastic mucoceles in our cohort reflects the pathway-based inclusion strategy rather than a neoplasm-enriched design. By starting from the mucocele label, we included benign endpoints that are excluded by design from neoplasm registries and many pathology-led neoplasm series. Clinical experience reports and broader reviews that include the mucinous spectrum similarly describe heterogeneity in final pathology and management across mucocele presentations [16,17,22,23].

A more relevant comparison across studies is the pathway consequence of histologic classification. Across modern frameworks, management is anchored in histology and a limited number of management-relevant pathology variables, particularly margin status and documentation of mucin beyond the appendix [5,6,7,8,9,10,11,12]. Our cohort is consistent with this framework: R1 margins and extra-appendiceal/serosal mucin occurred only in neoplastic lesions and were the features most closely associated with escalation to completion right hemicolectomy and structured oncologic follow-up [9,10,11,12].

Evidence for systemic therapy in appendiceal malignancy largely derives from registry-based analyses that include heterogeneous histologies and are often dominated by adenocarcinoma [25,26]. These data provide context for why chemotherapy may be considered in selected appendiceal cancers but should not be overlaid directly onto a LAMN/HAMN-focused pathway cohort. In our setting, the most defensible interpretation remains descriptive.

Overall, when interpreted within its intended frame, our cohort aligns with the literature in terms of rarity within appendectomy practice, heterogeneity of clinical entry points, and the central role of standardized histopathology in guiding escalation and follow-up [1,2,3,4,5,6,7,8,9,10,11,12,16,17,22,25,26]. The main point of difference, namely the higher frequency of non-neoplastic mucoceles than LAMN/HAMN, is consistent with the pathway-based design rather than in contradiction with neoplasm-enriched datasets.

### 4.7. Strengths and Limitations

A principal strength of this study is that it is anchored in routine surgical practice over a full decade, with clear denominators (2492 appendectomies; 704 right hemicolectomies). This framing is important because appendiceal neoplasms and mucinous appendiceal lesions are uncommon in appendectomy-based cohorts, and even large institutional series typically accumulate modest numbers over long intervals [2,3]. By using a mucocele-pathway entry point and then resolving diagnosis through histopathology, the cohort captures the clinical scenario faced preoperatively rather than reflecting a neoplasm-enriched registry population [1,5].

A second strength is the focus on management-relevant variables that are clinically interpretable and aligned with modern classification and reporting. The cohort shows how standardized histopathology terminology (LAMN/HAMN) and a limited set of pathology findings (R status; extra-appendiceal/serosal mucin) map to escalation decisions and follow-up intensity [5,6,7,8,9,10,11]. This is the main pathway-related contribution of the study: it does not attempt imaging accuracy estimates, but demonstrates how the mucocele label resolves into distinct endpoints with different management consequences [1,5,6,7,8].

The limitations primarily reflect design and data structure. First, this is a retrospective, single-center cohort with a small sample size (*n* = 18), which limits generalizability and precludes meaningful inferential analysis or multivariable modeling; therefore, the findings should be interpreted as descriptive and hypothesis-generating, although the sample size is consistent with the rarity of these lesions when denominators are considered [2,3]. Second, imaging was not uniform, and the analysis was not designed to evaluate CT diagnostic performance for histologic subtype; CT variables were used to describe pathway behavior rather than accuracy [1]. Third, absolute lymph node yield was not consistently reported in pathology records and was therefore not analyzed. Fourth, follow-up intensity differed by histologic endpoint: structured oncologic follow-up was concentrated in neoplastic cases, whereas many non-neoplastic mucoceles did not undergo formal surveillance, which limits direct outcome comparisons between groups and constrains interpretation of recurrence data in the non-neoplastic subgroup [24]. Finally, systemic therapy was administered in very few patients; registry-based chemotherapy evidence in appendiceal malignancies largely reflects heterogeneous histologies, often dominated by adenocarcinoma, and should not be extrapolated to infer treatment effect in a small LAMN/HAMN pathway cohort [25,26].

Despite these limitations, the study provides a practice-oriented description of how a common working label (“mucocele”) translates into modern histopathologic endpoints and how those endpoints inform escalation and follow-up under real-world conditions [5,6,7,8,9,10,11,12].

### 4.8. Practical Implications

A pathway paper should end with practical decision points that can be applied under real-world diagnostic uncertainty. In our cohort, the most useful operational message is that the “mucocele” label should trigger a structured sequence: safe index resection, standardized pathology reporting, and histopathology-driven escalation when indicated [9,10,11].

Preoperative suspicion, most often CT-based, should primarily guide operative planning rather than be used to infer etiology. CT is useful for recognizing a dilated mucus-filled appendix and for describing complications, but it cannot replace histopathology for differentiating non-neoplastic mucocele from LAMN/HAMN [1,5,6,7,8]. In practical terms, suspected mucocele should prompt rupture-avoidance planning and meticulous handling, consistent with guidance emphasizing safe resection and pathology-driven decision-making [9,10,11,14].

Within this pathway, index surgery functions as a risk-mitigation step performed before definitive classification is available. In our cohort, appendectomy was the dominant index operation, while primary right hemicolectomy occurred only in selected complex acute presentations, supporting a context-driven model rather than a uniform resection rule [11,13]. Because definitive classification often becomes available only after histopathology, documentation of intraoperative context remains important when pathology reveals LAMN/HAMN and postoperative planning is discussed in MDT settings [9,10,11].

The pivot point of the pathway is the pathology report. In our data, escalation clustered around a limited set of management-relevant variables: histologic subtype (LAMN/HAMN), margin status, and extra-appendiceal/serosal mucin [2,3,4,5,6,7,8]. Consistent reporting of these variables reduces ambiguity and may help avoid both overtreatment driven by label-based anxiety and undertreatment driven by incomplete risk documentation [2,3,4,5,24]. When microscopy figures are included, their value is greatest when linked directly to these decision nodes rather than presented as isolated illustrations [5,6,7,8].

Postoperative planning in our cohort was clearly histology-driven. Non-neoplastic mucoceles with negative margins and no extra-appendiceal mucin generally did not enter formal oncologic surveillance, reflecting routine practice patterns. In contrast, LAMN/HAMN cases concentrated follow-up intensity and escalation decisions, particularly when R1 margins or extra-appendiceal/serosal mucin were documented. This risk-adapted logic is consistent with guidance documents emphasizing MDT discussion and tailored surveillance when higher-risk features are present [9,10,11,24]. The R1 margin scenario in LAMN should be framed as a decision node rather than as a rigid rule; targeted institutional experience and literature review support individualized decisions rather than a universal requirement for additional surgery [12].

Contemporary reviews of mucinous appendiceal neoplasms emphasize heterogeneity across the mucinous spectrum and the need for consistent terminology and structured management [28,29,30]. Although molecular testing was not part of our cohort, molecular profiling work in high-grade mucinous neoplasms supports the broader concept that classification should remain explicit and structured rather than label-based [21].

## 5. Conclusions

The term “appendiceal mucocele” is clinically convenient but diagnostically incomplete. In this 10-year cohort, the same preoperative label encompassed two distinct endpoints—non-neoplastic mucoceles and neoplastic mucinous lesions (LAMN/HAMN)—with different management trajectories. Our findings suggest that the key step in this pathway is not the preoperative imaging impression, but the histopathology report, particularly margin status and the presence of extra-appendiceal/serosal mucin. In practical terms, suspected mucocele should be approached through a standardized pathway: safe index resection, structured pathology reporting using modern terminology, and risk-adapted escalation and follow-up when higher-risk features are present. Given the small sample size, these findings should be interpreted as descriptive and hypothesis-generating.

## Figures and Tables

**Table 1:** Baseline characteristics and key management features by histopathology group.

Variable	Total (*n* = 18)	Non-Neoplastic Mucocele (*n* = 10)	LAMN/HAMN (*n* = 8)	*p*-Value
Age, median (IQR), years	64.5 (62.5–66.5)	65.0 (63.3–66.8)	63.5 (57.8–65.5)	0.349
Female sex	12 (66.7%)	9 (90.0%)	3 (37.5%)	0.043
Emergency presentation	10 (55.6%)	2 (20.0%)	8 (100.0%)	0.001
CT performed	16 (88.9%)	10 (100.0%)	6 (75.0%)	0.183
Primary right hemicolectomy as index procedure	2 (11.1%)	0 (0.0%)	2 (25.0%)	0.183
Completion right hemicolectomy after appendectomy	4 (22.2%)	0 (0.0%)	4 (50.0%)	0.023
R1 margin after index procedure	4 (22.2%)	0 (0.0%)	4 (50.0%)	0.023
Extra-appendiceal/serosal mucin	4 (22.2%)	0 (0.0%)	4 (50.0%)	0.023
Systemic chemotherapy	2 (11.1%)	0 (0.0%)	2 (25.0%)	0.183
Documented recurrence	1 (5.6%)	NA	1 (12.5%)	NA

Abbreviations: CT—computed tomography; IQR, interquartile range; LAMN—low-grade appendiceal mucinous neoplasm; HAMN—high-grade appendiceal mucinous neoplasm; NA—not available (due to lack of structured follow-up in the non-neoplastic group). Note: *p*-values were calculated using Fisher’s exact test (categorical variables) and Mann–Whitney U test (continuous variables).

**Table 2:** Diagnostic pathway: CT use and report impression.

CT Status/Report Impression	Total (*n* = 18)	Non-Neoplastic (*n* = 10)	LAMN/HAMN (*n* = 8)
CT performed	16 (88.9%)	10 (100.0%)	6 (75.0%)
No CT	2 (11.1%)	0 (0.0%)	2 (25.0%)
**CT impression (all cases, mutually exclusive)**			
Mucocele / suspected mucinous lesion	12 (66.7%)	8 (80.0%)	4 (50.0%)
Appendicitis/perforation (no mucocele suspicion)	2 (11.1%)	0 (0.0%)	2 (25.0%)
Obstruction	2 (11.1%)	2 (20.0%)	0 (0.0%)
No CT (as above)	2 (11.1%)	0 (0.0%)	2 (25.0%)

Abbreviations: CT—computed tomography; LAMN—low-grade appendiceal mucinous neoplasm; HAMN—high-grade appendiceal mucinous neoplasm.

**Table 3:** Surgical management: index procedure and escalation.

Surgical Variable	Total (*n* = 18)	Non-Neoplastic (*n* = 10)	LAMN/HAMN (*n* = 8)
**Index procedure**			
Appendectomy—open	15 (83.3%)	9 (90.0%)	6 (75.0%)
Appendectomy—laparoscopic	1 (5.6%)	1 (10.0%)	0 (0.0%)
Primary right hemicolectomy	2 (11.1%)	0 (0.0%)	2 (25.0%)
Completion right hemicolectomy after appendectomy	4 (22.2%)	0 (0.0%)	4 (50.0%)
Time to completion hemicolectomy, weeks (range)	4–6	—	4–6

Abbreviations: LAMN—low-grade appendiceal mucinous neoplasm; HAMN—high-grade appendiceal mucinous neoplasm.

**Table 4:** Histopathology and actionable findings.

Variable	Total (*n* = 18)	Non-neoplastic (*n* = 10)	LAMN/HAMN (*n* = 8)
**Final histopathology**			
Non-neoplastic mucocele	10 (55.6%)	10 (100.0%)	0 (0.0%)
LAMN	4 (22.2%)	0 (0.0%)	4 (50.0%)
HAMN	1 (5.6%)	0 (0.0%)	1 (12.5%)
Combined LAMN/HAMN	3 (16.7%)	0 (0.0%)	3 (37.5%)
**Actionable pathology**			
R1 at index procedure	4 (22.2%)	0 (0.0%)	4 (50.0%)
Extra-appendiceal/serosal mucin	4 (22.2%)	0 (0.0%)	4 (50.0%)
pT4a documented	1 (5.6%)	0 (0.0%)	1 (12.5%)

Abbreviations: LAMN—low-grade appendiceal mucinous neoplasm; HAMN—high-grade appendiceal mucinous neoplasm.

## Data Availability

The original contributions presented in the study are included in the article; further inquiries can be directed to the corresponding author.

## Abbreviations

The following abbreviations are used in this manuscript:

CT	Computed tomography
HAMN	High-grade appendiceal mucinous neoplasm
IQR	Interquartile range
LAMN	Low-grade appendiceal mucinous neoplasm
MDT	Multidisciplinary team
R0	Microscopically negative resection margin
R1	Microscopically positive resection margin
pT4a	Pathologic tumor stage T4a
WHO	World Health Organization
